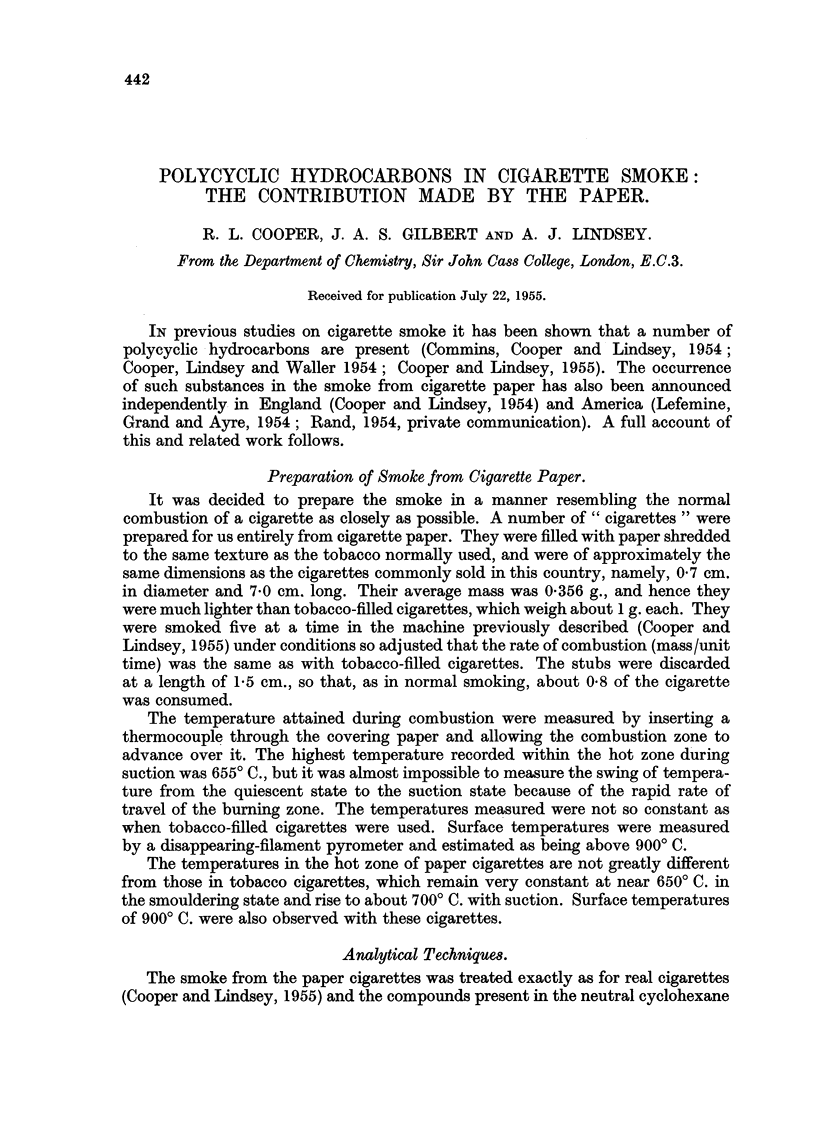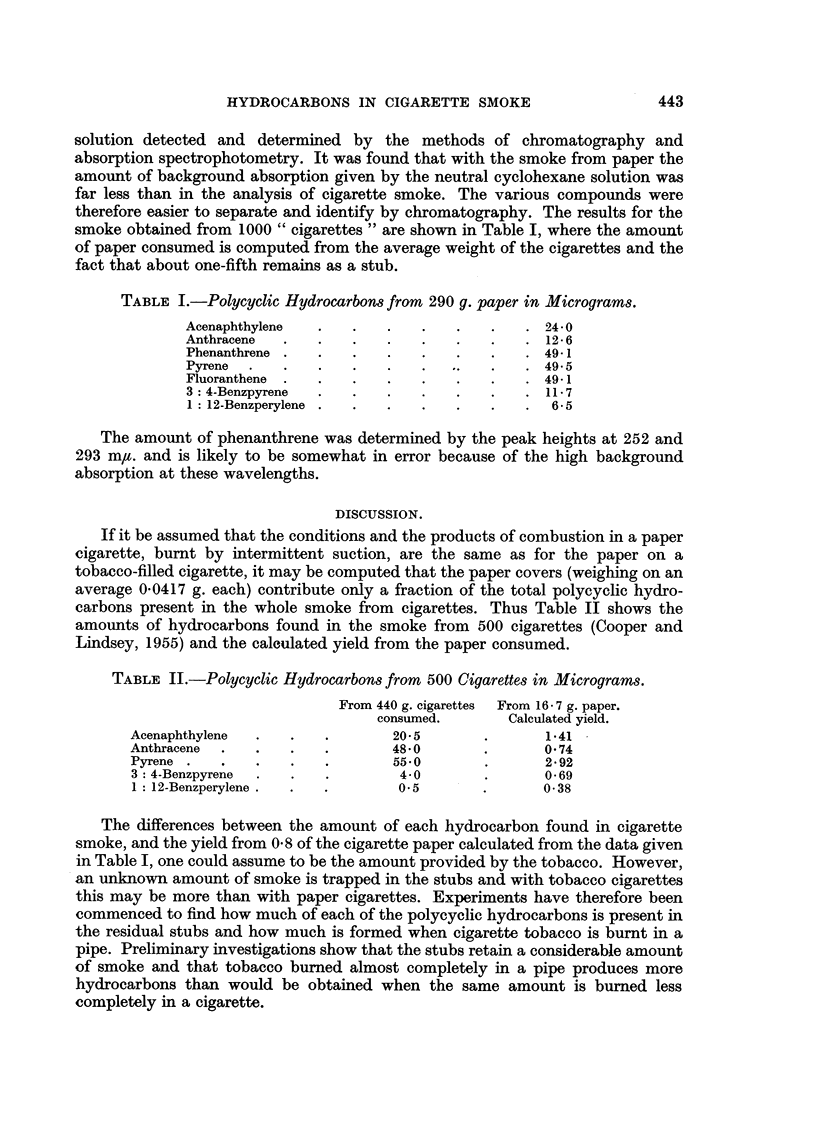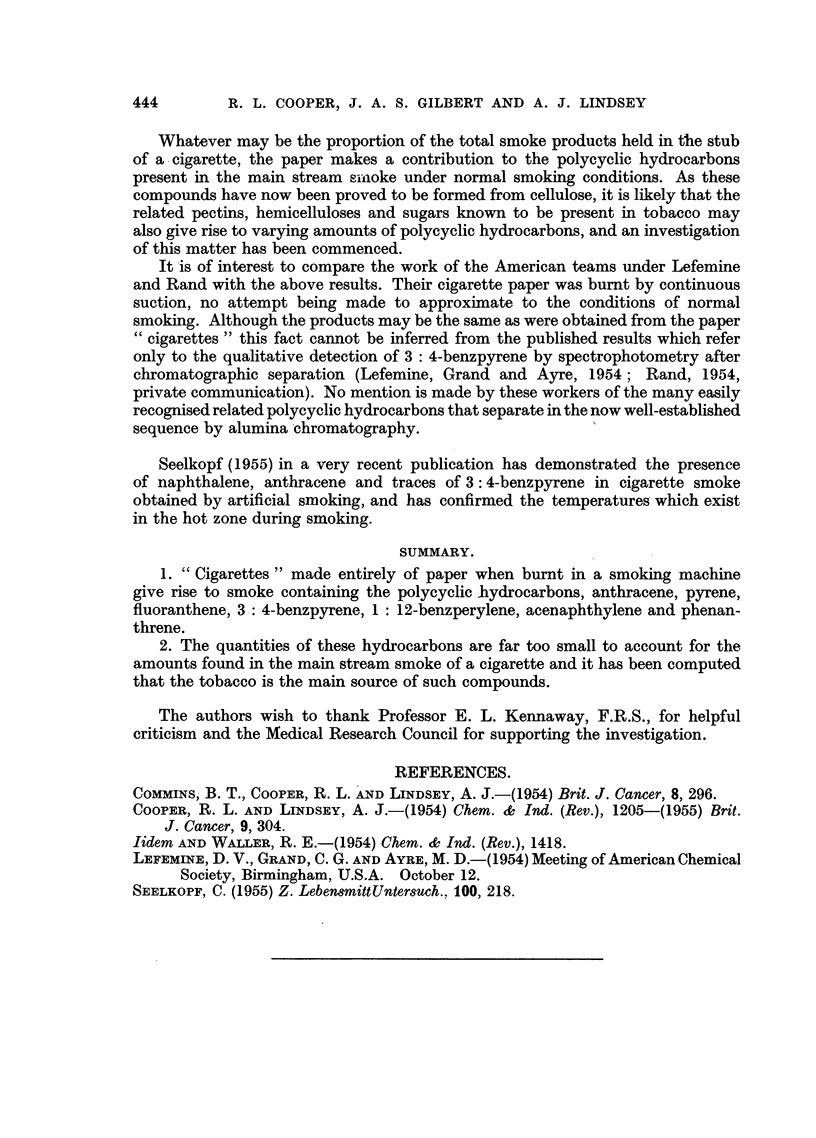# Polycyclic Hydrocarbons in Cigarette Smoke: The Contribution made by the Paper

**DOI:** 10.1038/bjc.1955.43

**Published:** 1955-09

**Authors:** R. L. Cooper, J. A. S. Gilbert, A. J. Lindsey


### POLYCYCLIC HYDROCARBONS IN CIGARETTE SMOKE: THE CONTRIBUTION MADE BY THE PAPER.

R. L. COOPER, J. A. S. GILBERT AND A. J. LINDSEY.

*From the Department of Chemistry, Sir John Cass College, London, E.C.3.*

Received for publication July 22, 1955.

IN previous studies on cigarette smoke it has been shown that a number of polycyclic hydrocarbons are present (Commins, Cooper and Lindsey, 1954; Cooper, Lindsey and Waller 1954; Cooper and Lindsey, 1955). The occurrence of such substances in the smoke from cigarette paper has also been announced independently in England (Cooper and Lindsey, 1954) and America (Lefemine, Grand and Ayre, 1954; Rand, 1954, private communication). A full account of this and related work follows.

### *Preparation of Smoke from Cigarette Paper.*

It was decided to prepare the smoke in a manner resembling the normal combustion of a cigarette as closely as possible. A number of " cigarettes " were prepared for us entirely from cigarette paper. They were filled with paper shredded to the same texture as the tobacco normally used, and were of approximately the same dimensions as the cigarettes commonly sold in this country, namely, 0·7 cm. in diameter and 7·0 cm. long. Their average mass was 0·356 g., and hence they were much lighter than tobacco-filled cigarettes, which weigh about 1 g. each. They were smoked five at a time in the machine previously described (Cooper and Lindsey, 1955) under conditions so adjusted that the rate of combustion (mass/unit time) was the same as with tobacco-filled cigarettes. The stubs were discarded at a length of 1·5 cm., so that, as in normal smoking, about 0·8 of the cigarette was consumed.

The temperature attained during combustion were measured by inserting a thermocouple through the covering paper and allowing the combustion zone to advance over it. The highest temperature recorded within the hot zone during suction was 655° C., but it was almost impossible to measure the swing of temperature from the quiescent state to the suction state because of the rapid rate of travel of the burning zone. The temperatures measured were not so constant as when tobacco-filled cigarettes were used. Surface temperatures were measured by a disappearing-filament pyrometer and estimated as being above 900° C.

The temperatures in the hot zone of paper cigarettes are not greatly different from those in tobacco cigarettes, which remain very constant at near 650° C. in the smouldering state and rise to about 700° C. with suction. Surface temperatures of 900° C. were also observed with these cigarettes.

### *Analytical Techniques.*

The smoke from the paper cigarettes was treated exactly as for real cigarettes (Cooper and Lindsey, 1955) and the compounds present in the neutral cyclohexane solution detected and determined by the methods of chromatography and absorption spectrophotometry. It was found that with the smoke from paper the amount of background absorption given by the neutral cyclohexane solution was far less than in the analysis of cigarette smoke. The various compounds were therefore easier to separate and identify by chromatography. The results for the smoke obtained from 1000 " cigarettes " are shown in Table I, where the amount of paper consumed is computed from the average weight of the cigarettes and the fact that about one-fifth remains as a stub.

TABLE I.—*Polycyclic Hydrocarbons from 290 g. paper in Micrograms.*

| | |
|---|---|
| Acenaphthylene | 24·0 |
| Anthracene | 12·6 |
| Phenanthrene | 49·1 |
| Pyrene | 49·5 |
| Fluoranthene | 49·1 |
| 3 : 4-Benzpyrene | 11·7 |
| 1 : 12-Benzperylene | 6·5 |

The amount of phenanthrene was determined by the peak heights at 252 and 293 m$\mu$. and is likely to be somewhat in error because of the high background absorption at these wavelengths.

DISCUSSION.

If it be assumed that the conditions and the products of combustion in a paper cigarette, burnt by intermittent suction, are the same as for the paper on a tobacco-filled cigarette, it may be computed that the paper covers (weighing on an average 0·0417 g. each) contribute only a fraction of the total polycyclic hydrocarbons present in the whole smoke from cigarettes. Thus Table II shows the amounts of hydrocarbons found in the smoke from 500 cigarettes (Cooper and Lindsey, 1955) and the calculated yield from the paper consumed.

TABLE II.—*Polycyclic Hydrocarbons from 500 Cigarettes in Micrograms.*

| | From 440 g. cigarettes consumed. | From 16·7 g. paper. Calculated yield. |
|---|---|---|
| Acenaphthylene | 20·5 | 1·41 |
| Anthracene | 48·0 | 0·74 |
| Pyrene | 55·0 | 2·92 |
| 3 : 4-Benzpyrene | 4·0 | 0·69 |
| 1 : 12-Benzperylene | 0·5 | 0·38 |

The differences between the amount of each hydrocarbon found in cigarette smoke, and the yield from 0·8 of the cigarette paper calculated from the data given in Table I, one could assume to be the amount provided by the tobacco. However, an unknown amount of smoke is trapped in the stubs and with tobacco cigarettes this may be more than with paper cigarettes. Experiments have therefore been commenced to find how much of each of the polycyclic hydrocarbons is present in the residual stubs and how much is formed when cigarette tobacco is burnt in a pipe. Preliminary investigations show that the stubs retain a considerable amount of smoke and that tobacco burned almost completely in a pipe produces more hydrocarbons than would be obtained when the same amount is burned less completely in a cigarette.

Whatever may be the proportion of the total smoke products held in the stub of a cigarette, the paper makes a contribution to the polycyclic hydrocarbons present in the main stream smoke under normal smoking conditions. As these compounds have now been proved to be formed from cellulose, it is likely that the related pectins, hemicelluloses and sugars known to be present in tobacco may also give rise to varying amounts of polycyclic hydrocarbons, and an investigation of this matter has been commenced.

It is of interest to compare the work of the American teams under Lefemine and Rand with the above results. Their cigarette paper was burnt by continuous suction, no attempt being made to approximate to the conditions of normal smoking. Although the products may be the same as were obtained from the paper " cigarettes " this fact cannot be inferred from the published results which refer only to the qualitative detection of 3 : 4-benzpyrene by spectrophotometry after chromatographic separation (Lefemine, Grand and Ayre, 1954 ; Rand, 1954, private communication). No mention is made by these workers of the many easily recognised related polycyclic hydrocarbons that separate in the now well-established sequence by alumina chromatography.

Seelkopf (1955) in a very recent publication has demonstrated the presence of naphthalene, anthracene and traces of 3 : 4-benzpyrene in cigarette smoke obtained by artificial smoking, and has confirmed the temperatures which exist in the hot zone during smoking.

### SUMMARY.

1. " Cigarettes " made entirely of paper when burnt in a smoking machine give rise to smoke containing the polycyclic hydrocarbons, anthracene, pyrene, fluoranthene, 3 : 4-benzpyrene, 1 : 12-benzperylene, acenaphthylene and phenanthrene.

2. The quantities of these hydrocarbons are far too small to account for the amounts found in the main stream smoke of a cigarette and it has been computed that the tobacco is the main source of such compounds.

The authors wish to thank Professor E. L. Kennaway, F.R.S., for helpful criticism and the Medical Research Council for supporting the investigation.

### REFERENCES.

Commins, B. T., Cooper, R. L. and Lindsey, A. J.—(1954) *Brit. J. Cancer*, **8**, 296.
Cooper, R. L. and Lindsey, A. J.—(1954) *Chem. & Ind.* (*Rev.*), 1205—(1955) *Brit. J. Cancer*, **9**, 304.
*Iidem* and Waller, R. E.—(1954) *Chem. & Ind.* (*Rev.*), 1418.
Lefemine, D. V., Grand, C. G. and Ayre, M. D.—(1954) Meeting of American Chemical Society, Birmingham, U.S.A. October 12.
Seelkopf, C. (1955) *Z. LebensmittUntersuch.*, **100**, 218.